# 

**Published:** 1892-11-26

**Authors:** 


### CONTENTS.

**Moral Antiseptics** . . . . . 129

**Passing Topics.** The "Ordeal of Pauperism" . . . . 131

**Diet in Disease** By Mrs. Ernest Hart. VI.—Water—Salt . 131

**Medical History from the Earliest Times.** By E. T. Withington, M.A., M.B.Oxon. XXXIII.—General Survey of Arabic Medicine . . . . . 133

**The Hospital Clinic—**

Royal Infirmary, Edinburgh.—The Treatment of Tubercular Disease of the Knee-Joint . . . . 137

St. Bartholomew's Hospital.—The Treatment of Colles's Fracture . . . . . 138

Central London Throat and Ear Hospital.—The Treatment of Post Nasal Adenoids . . . . . 139

The Practitioners' Bookshelf.—Medical Microscopy—Artificial Feeding and Food Disorders of Children . . . . 140

New Drugs, Appliances, and Things Medical.—Wholesome Bread—A New Cocoa . . . . . 140

**The Worth of Inflammation** . . . . . 132

**The Attitude of Great Writers Towards Disease.** XVI. —Boccaccio . . . . . 134

**Annotations.**—Is the London Medical School a Failure?—"Long Runs"—Riotous Living at Kidderminster Workhouse . . 135

**Notes and News** . . . . . 136

**In Memoriam.**—John Charles Steele, M.D. . . . . 140

**Books Received** . . . . . 140

**The Institutional Workshop—**

Hospital Construction.—Opening of the New Medical Wing University College, Bristol, by Sir Andrew Clark, Bart., M.D., LL.D., F.R.S. . . . . . 141

Hospital Meetings.—British Home for Incurables, Clapham Rise . . . . . 143

Construction Notes . . . . . 144

**Scraps and Gleanings** . . . . . 144

**The Student of Medicine.**—Notes from the Schools—Vacancies.

### EXTRA SUPPLEMENT.—THE NURSING MIRROR.

**En Passant.**—Metropolitan Asylums Board—St. John's Maternity Home—Home for Nurses, Cambridge—The Pinnacle of Perfection—Belfast Nurses—Kingston Board of Guardians—Nursing in India—The Want of Money . . . xlix

**Lectures for Asylum Attendants.** By William Harding, M.B. IX.—Epileptics . . . . . l

**The Registration of Nurses and the R.B.N.A.**—Application to the Privy Council . . . . . li

**Appointments** . . . . . lviii

**Notes on Novelties.**—The Sanitary Wood Wool Company—Shetland Hand-Knit Goods . . . . . lix

**Everybody's Opinion.**—Examination Papers—Nurses Fees—Nursing in Bombay . . . . . lix

**Notes and Queries Wants and Workers** . . . . lxi

**Minor Appointments—Where to Go** . . . . lx

**The Muses' Looking Glass.**—"La Cité de M sère" . . lx

